# The Pharmaceutical and Pharmacological Potential Applications of Bilosomes as Nanocarriers for Drug Delivery

**DOI:** 10.3390/molecules30051181

**Published:** 2025-03-06

**Authors:** Darko Mitrović, Dragana Zaklan, Maja Đanić, Bojan Stanimirov, Karmen Stankov, Hani Al-Salami, Nebojša Pavlović

**Affiliations:** 1 Department of Pharmacy, Faculty of Medicine, University of Novi Sad, 21000 Novi Sad, Serbia; 904004d22@mf.uns.ac.rs (D.M.); dragana.zaklan@mf.uns.ac.rs (D.Z.); 2 Department of Pharmacology, Toxicology and Clinical Pharmacology, Faculty of Medicine, University of Novi Sad, 21000 Novi Sad, Serbia; maja.djanic@mf.uns.ac.rs; 3 Department of Biochemistry, Faculty of Medicine, University of Novi Sad, 21000 Novi Sad, Serbia; bojan.stanimirov@mf.uns.ac.rs (B.S.); karmen.stankov@mf.uns.ac.rs (K.S.); 4 The Biotechnology and Drug Development Research Laboratory, Curtin Medical School and Curtin Health Innovation Research Institute, Curtin University, Perth, WA 6845, Australia; hani.al-salami@curtin.edu.au; 5 UWA Medical School, University of Western Australia, Perth, WA 6009, Australia

**Keywords:** bilosomes, lipid vesicles, bioavailability, nanotechnology, liposomes, bile acids

## Abstract

Nano-drug delivery systems provide targeted solutions for addressing various drug delivery challenges, leveraging nanotechnology to enhance drug solubility and permeability. Liposomes, explored for several decades, face hurdles, especially in oral delivery. Bile-acid stabilized vesicles (bilosomes) are flexible lipid vesicles, composed of phospholipids or other surfactants, along with amphiphilic bile salts, and they show superior stability and pharmacokinetic behavior in comparison to conventional vesicular systems (liposomes and niosomes). Bilosomes enhance skin penetration, fluidize the stratum corneum, and improve drug stability. In oral applications, bilosomes overcome drawbacks, offering improved bioavailability, controlled release, and reduced side effects. Vaccines using bilosomes demonstrate efficacy, and bilosomes for intranasal, inhalation, ocular, and buccal applications enhance drug delivery, offering targeted, efficient, and controlled activities. Formulations vary based on active substances and optimization techniques, showcasing the versatility and potential of bilosomes across diverse drug delivery routes. Therefore, the aim of this comprehensive review was to critically explore the state-of-the-art of bilosomes in drug delivery and potential therapeutic applications.

## 1. Introduction

Nanotechnology is a highly attractive and rapidly expanding area of scientific research and development, due to its immensely broad range of applications and advancements that can be achieved by it, as well as noteworthy economic viability. Although still a relatively novel and ever-developing concept, nanotechnology has already made a momentous impact in the fields of medical and pharmaceutical sciences. In this sense, one of the most promising and extensively studied applications is the design of advanced drug delivery systems, with the aim of circumventing the limitations of conventional pharmaceutical dosage forms and ultimately improving the biopharmaceutical profile and bioavailability of both traditional and novel therapeutic compounds [1]. Nanosizing is a pharmaceutical process that allows achieving the nanoparticles typically ranging in size between 1 nm and 100 nm. Exhibiting unique and diverse structural, chemical, mechanical, thermal, electrical, magnetic, optical, and biological properties, nanoparticulate drug delivery systems offer advantages over traditional dosage forms attributable to their distinctive characteristics. Thus, such nanostructures could be employed as drug carriers and manipulated to achieve precise delivery to specific tissues, cells, or cell compartments, or release the cargo in a controlled manner [2,3,4,5]. Furthermore, new drug delivery systems (NDDS) represent one of the most crucial strategies for overcoming issues related to drug bioavailability. Bioavailability refers to the speed and extent to which a drug is absorbed from a dosage form and becomes available to the target tissue after administration. Many of the contemporary drugs in use exhibit poor bioavailability, necessitating administration in higher doses as only a minor fraction of the applied dose is absorbed into the systemic circulation and reaches the intended site. This may result in undesired side effects as well as drug wastage accompanied by economic consequences. Amid the various traditional and innovative approaches developed with the primary aim of improving the solubility and permeability of drugs with low bioavailability, vesicular drug delivery systems, evolving from the initially developed liposomes to more advanced structures such as bilosomes, have drawn much interest due to their outstanding merits and advantages over conventional alternatives [6,7,8].

Considering that rapidly advancing nanotechnology undoubtedly represents the future of drug delivery and offers unprecedented therapeutic breakthroughs, as well as the vast potential of bilosomes as highly promising lipid-based nanosystems, this study aimed to highlight and explore the fundamental information related to bilosome structure, types, and production technologies used, along with their application in the design and development of novel pharmaceutical dosage forms and prospective routes of drug administration for improved therapeutic efficacy. The novelty of this study is in the comprehensive review of bilosomes used through different administration routes, as well as in the identification of optimal bilosome formulation compositions.

## 2. Literature Search Methodology

This review presents a comprehensive literature search of relevant publications up to August 2024, conducted across several scientific databases, including PubMed, Scopus, and Web of Science. The key search terms included “bilosomes”, “bile-salt stabilized liposomes”, “bile-salt stabilized niosomes”, “drug delivery”, and “nanovesicles”, alone or in combination. Only English-language original studies were considered for inclusion, while non-research publications were excluded. The process of literature search, screening, and data extraction was carried out by two independent researchers, with any discrepancies resolved through consensus. A total of 93 original research articles were included in the review and categorized based on the administration route of the active substance.

## 3. Nanovesicular Drug Delivery Systems: A Comparative Short Review

Nano-based vesicular drug delivery systems are characterized by a vesicular structure comprising one or several concentric or continuous bilayers formed as a result of the self-assembly of amphiphilic building blocks in the aqueous medium. Vesicle-forming materials essentially determine physicochemical properties, drug delivery performance, and efficacy, as well as the in vivo fate of the vehicle. The broad spectrum of highly tunable natural and artificial materials provides numerous opportunities to design versatile and customized vesicular drug delivery systems, also regarded as “rebirth systems”, due to the fact that every novel formulation offers optimized or superior performances [6,9].

Liposomes have been well developed and characterized and are one of the first known nanocarrier vesicles, introduced and described by Bangham in the early 1960s. Since then, they have been extensively studied as high-potential delivery vehicles targeting specific locations in the body and successfully utilized in the pharmaceutical and cosmetic industries. Liposomes are nearly spherical, closed vesicles composed of one or more concentric phospholipid bilayers arranged around a central aqueous core. Thus, their biochemical structure closely resembles normal human cell membranes and grants favorable biocompatibility and safety profile. The size of liposomes can range from 20 nm in the smallest vesicles to 1 mm or even more in liposomes visible under a light microscope. The specific amphipathic nature of their constituents allows for lipid-soluble drugs to be embedded in hydrophobic lipid bilayers and water-soluble drugs in the inner aqueous phase. Furthermore, liposomes can incorporate peptides and small proteins at the lipid–water interface. The release of drugs from liposomes, as well as their stability and pharmacokinetic profiles, depends on the composition, size, surface charge, and solubility of the drug. Hence, adequately engineered liposomes provide protection to entrapped therapeutic agents against deterioration in vivo, prolong the drug’s half-life, regulate and navigate drug delivery to specific sites and ultimately reduce hazardous side effects while enhancing treatment outcomes. Liposomal formulations of various drugs have shown a significant increase in therapeutic activity compared to non-liposomal counterparts, due to the improved stability and biodistribution of the therapeutic agents. However, conventional liposomes suffer from several indisputable impediments, including susceptibility to degradation in the gastrointestinal environment and clearance by the reticuloendothelial system resulting in poor plasma stability, as well as physicochemical instability in aqueous dispersions, cargo leakage, and challenging transfer to large-scale production and clinical usage due to batch-to-batch variations. Numerous strategies have been developed and applied in an attempt to overcome the aforementioned limitations and enhance the performance of liposomal formulations. Recent advances in this area include modulation of lipid components, surface coating, thickening of the interior aqueous phase, absorption enhancement by employing coating polymers with mucoadhesive characteristics, various absorption enhancers, or specific ligands for facilitated targeting, for instance. Furthermore, other amphiphilic alternatives to phospholipids have been widely studied to obtain vesicular carriers with improved functionality. In this sense, niosomes emerged as highly attractive alternatives to liposomes, especially as carriers of poorly absorbable therapeutics [3,10,11,12].

Niosomes are unilamellar or multilamellar vesicular structures made of non-ionic surfactants and a hydration medium that efficiently load both hydrophilic and hydrophobic therapeutic agents. Since non-ionic surfactants are easily derivatized and versatile in nature, not only are niosomes characterized by cost efficiency, but also improved stability, biocompatibility, and safety. In addition, niosomes are usually stabilized by the addition of lipid components, namely cholesterol, which positively impacts the rigidity, permeability, leakage, and entrapment efficacy of the vesicle. However, niosomes still suffer from certain drawbacks, including aggregation and fusion of vesicles, drug leakage, and hydrolysis, as well as instability in the harsh gastrointestinal environment, analogous to liposomes [13,14,15,16]. Soon after their discovery, liposomes were investigated for the oral delivery of peptides and proteins. Liposomes stabilize proteins in the gastrointestinal (GI) tract to some extent but also demonstrate instability after oral administration due to the action of bile salts, pancreatic enzymes, and the acidic environment in the stomach. Bile salts initially destabilize liposome membranes; however, it has been demonstrated that prior incorporation of bile salts into liposomal bilayers exerts a stabilizing effect and prevents their further degradation. Therefore, it is indicated that vesicles containing bile salts may act as more stable carriers than conventional liposomes and niosomes, facilitating transmembrane transport and drug absorption. As a result, bile salt-stabilized nanovesicular systems, known as bilosomes, emerged as unique, novel, and promising drug delivery vehicles combining the merits and overcoming drawbacks of aforementioned vesicular carriers, making them better for oral and transdermal delivery than liposomes or niosomes [10,11,17].

## 4. Bilosomes and Bilosome Technologies

### 4.1. Bilosome Formulations—Composition and Types

**Figure 1:**
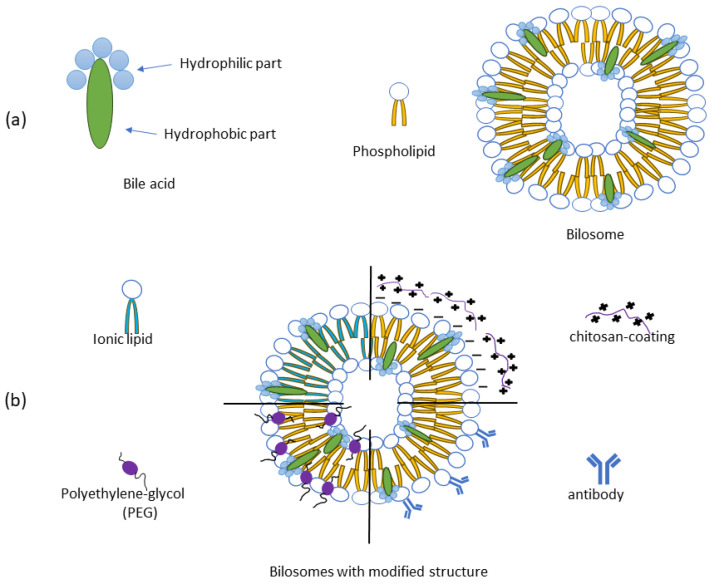
Schematic representation of bilosome structure: (**a**) typical bilosome structure; (**b**) bilosomes with surface modifications.

The discovery of liposomes more than 50 years ago gave significant rise to lipid-based drug delivery research and in vitro and in vivo evaluation of novel formulations. Over time, liposomes have proved to be promising drug delivery platforms, and several liposomal formulations have been successfully marketed and established in clinical practice. However, successful cases are mainly limited to the parenteral route of drug administration. Although it is one of the most effective routes of drug delivery, some of its major drawbacks include patient discomfort and consequently low compliance [11,18]. Both liposomes and niosomes have been tested as vehicles for oral drug delivery, but the issue of their instability in the hostile gastrointestinal environment and unsatisfactory permeability across epithelia remains a concern [19]. In this context, vesicles containing bile salts, known as bilosomes, have demonstrated superior performance [20]. Bilosomes are closed, double-layered vesicular carriers containing lipids, non-ionic surfactants, and bile salts as structural elements, mixed in different ratios to prepare the optimal vehicle for the transport of encapsulated drugs via the targeted route of administration. Thus, bilosomes can be developed from either liposomes (bile salt-liposomes) or niosomes (bile salt-niosomes). Their size ranges from 5–200 nm, often exhibiting a spherical shape and existing in the form of single or multi-layered vesicles (Figure 1a) [21,22,23].

The most commonly utilized lipid components comprising bilosomes are phospholipids, cholesterol, and lecithin. Phospholipids are characterized by amphipathic properties, due to the presence of a hydrophilic phosphate group and a hydrophobic acyl chain in the structure. Consequently, they exert excellent biocompatibility, self-assembling, emulsifying, and wetting properties. A whole range of phospholipids varying in composition is available for the synthesis of vesicular nanocarriers. Cholesterol has a significant impact on the physicochemical characteristics and stability of bilosomes. Being an amphiphilic molecule, cholesterol can be inserted into the membrane with hydroxyl groups oriented toward the aqueous core and aliphatic chains aligned parallel to the acyl chains, which ultimately increases the rigidity and stability of the bilosomal membrane and reduces the leakage of entrapped water-soluble substances. In the same vein, lecithin may support the stability of the vehicle, in addition to adjusting the solubility of the entrapped drug [21,24,25,26]. Non-ionic surfactants are notable components of both niosomes and bilosomes, due to their amphiphilicity, low toxicity, stability, and biocompatibility. They are less irritative and hemolytic in comparison with their anionic, cationic, and amphoteric counterparts. Furthermore, they have solubilizing, emulsifying, wetting, and permeability-enhancing properties. Hydrophilic–lipophilic balance (HLB), critical packing parameter and gel liquid transmission temperature immensely impact the selection of non-ionic surfactant for bilosome synthesis, as these parameters determine the physicochemical features, drug entrapment efficacy, and in vivo fate of the carrier. Among the various alternatives, the most extensively utilized are polysorbates, alkyl esters, and alkyl ethers [21,26].

Various surface modifications of bilosomes have been recently investigated to improve their targeting efficiency against a range of diseases. Those modifications most commonly involve polymer coatings, e.g., electrostatic interactions between the negatively charged surface of the bilosomes and the positively charged amino acid groups of the mucoadhesive polymer chitosan. Coating with polyethylene glycol (PEG), on the other hand, increases circulation time and reduces immune recognition. The bilosome surface can be functionalized with antibodies as well, which enables targeted drug delivery to specific cells or tissues. Eventually, the incorporation of cationic lipids, such as DOTAP, instead of phospholipids, improves interaction with negatively charged cell membranes (Figure 1b) [24,27,28].

Over the last decade, the scientific community’s attention has been focused on bile acids and their salts in the context of drug transport systems development, their role as drug carriers, and potential application in the context of nanomedicine, due to their favorable biological compatibility and safety profile. Bile acids are amphiphilic steroidal bio-surfactants and the major organic solute in bile, synthesized from cholesterol in the liver. Bile acids are characterized by a distinguished chemical structure, due to the presence of a large, rigid, and planar hydrophobic steroid core with hydroxyl groups varying in number, position, and orientation, along with a flexible acidic side chain. The rigidity of the structure as well as the complex distribution of hydrophilic and hydrophobic structural components impact the surface activity, specific self-assembly behavior, and interfacial properties of bile acids [23,29,30,31]. Thus, the primary physiological functions of endogenous bile acids include emulsification and absorption of dietary lipids, liposoluble vitamins, and other lipophilic nutrients, as well as maintenance of healthy microbiota, endocrine functions, and immune response [32]. In the pharmaceutical domain, bile acids and their salts have been employed based on two distinct concepts. The first concept leverages the metabolic and homeostatic functions of these biomolecules in disease modulation, while the second exploits their potential to modify drug delivery characteristics, recently incorporating nanotechnology [33,34]. Bile acids and their salts may ameliorate the bioavailability of therapeutic agents whose poor aqueous solubility or low membrane permeability are absorption-limiting factors. The proposed mechanisms include solubilization of hydrophobic drugs, promotion of their chemical and enzymatic stability, ion-pairing, increase in the fluidity and permeability of biological membranes, membranolysis at concentrations above critical micellar concentration, formation of reverse micelles, tight junction opening, as well as a mucolytic effect. Consequently, bile acids have been widely investigated as permeation enhancers in oral, transdermal, pulmonary, nasal, buccal, mucosal, rectal, ocular, and blood–brain barrier delivery [35,36]. Furthermore, bile acids are promising candidates for use in nanotechnology and bioengineering as drug carriers and formulation enhancers in the form of chemical conjugates, complexes, mixed micelles, and bilosomes [33,34,37].

Bilosomes are innovative drug delivery carriers comprising bile salts incorporated into the liposomal or niosomal membrane. Conventional vesicles are known to undergo enzymatic degradation in the gut. However, the incorporation of bile salts into the formulation can help stabilize the vesicle and protect it from the demoting effects of endogenous bile acids by causing repulsion. Thus, bilosomes have been found to enhance the therapeutic efficacy of drugs due to their increased stability in the gastrointestinal tract, which is additionally fortified by their nanometric size, hydrophobicity, and negative surface charge. Moreover, bilosomes can be readily absorbed through the small intestine into the portal circulation, which may add to the increased bioavailability of the entrapped therapeutic [21]. Additional points of strength include biocompatibility and a favorable safety profile. Although they were initially developed to alleviate oral drug delivery, bilosomes have been evaluated for drug delivery via other routes as well. The transition into wider application and potentially into clinical practice may be supported by technical feasibility to scale up the fabrication of bilosomes [33,38].

### 4.2. Methods of Preparation and Industrial Potential of Bilosomes

**Figure 2:**
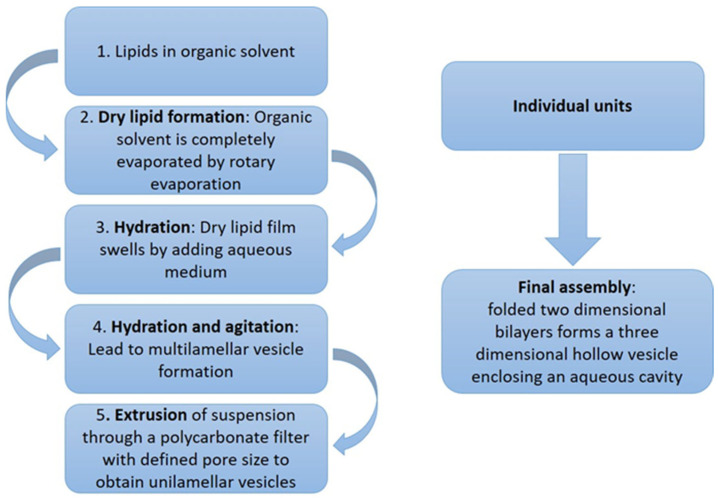
Schematic representation of the thin film hydration method for bilosome formation.

Various methods are employed to obtain these nanocarriers. The thin lipid film hydration method, known as the Bangham method, was initially used for bilosome preparation (Figure 2) [39,40].

As the industrial production of nanoformulations has become a reality and a necessity, new techniques for producing larger quantities of bilosomes have been developed and tested. These include heating techniques, spray drying, freeze-drying (lyophilization), supercritical reverse-phase evaporation, microwave irradiation, and several highly attractive modified ethanol injection techniques, such as cross-injection, microfluidization, and membrane contactor application techniques [22].

Currently, bilosomes are still primarily in the research and development phase, and there are no available bilosome-based commercial products. Most existing bilosome-based patents focus on oral vaccine delivery. It should be emphasized that the high cost of bilosome synthesis requires further evaluation to facilitate their large-scale application in the pharmaceutical industry. The industrial scale-up considerations in manufacturing bilosomes need to be optimized to enhance encapsulation efficiency and produce a cost-effective product. Additionally, maintaining bilosome stability often demands specific storage conditions, such as refrigeration, which can be challenging in certain environments [24].

Like other vesicular drug carriers, bilosomes require robustness and reproducibility for successful industrial scale-up. Quality by Design (QbD), as a systematic approach to pharmaceutical development that emphasizes designing and controlling manufacturing processes to ensure product quality from the outset, along with Formulation by Design (FbD), as a specialized application of QbD focused on the rational design of pharmaceutical formulations, are nowadays an essential part of drug development and manufacturing process, since they are incorporated in the official guidelines of regulatory authorities. The first step in the QbD process is defining formulation objectives, i.e., establishing objectives for drug delivery. In this step, many critical quality attributes (CQAs) or response variables, which practically characterize the objectives, are assigned for this purpose [41]. Therefore, the initial step is to identify potential CQAs of the product, based on the quality target product profile (QTPP), by means of a risk assessment [42].

**Figure 3:**
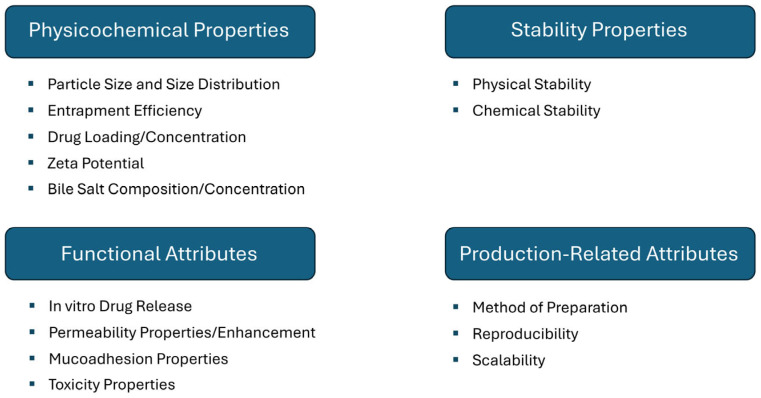
Critical quality attributes (CQAs) in bilosomes development.

CQAs of bilosomes refer to the key physicochemical and biological properties that affect their quality, safety, and efficacy, and they must be cautiously controlled during formulation and manufacturing. It was shown that average particle size, encapsulation efficiency, and vesicular drug concentration are the parameters that most influence the drug delivery system, mostly through their impact on cellular uptake and biodistribution. However, some other properties may significantly impact the quality or efficacy of bilosomes (Figure 3), such as physicochemical properties (zeta potential that impacts electrostatic stability or bile salt type/concentration influencing resistance to enzymatic degradation), stability properties (vesicle integrity under different storage conditions and degradation of bilosome components or the encapsulated drug), functional attributes (drug release kinetics, the ability of bilosomes to improve drug transport across biological barriers, mucoadhesion that ensures sufficient residence time at the absorption site, and biocompatibility and toxicity), and the process-related attributes (e.g., method of preparation that affects particle characteristics). Controlling these CQAs is essential to ensure that bilosomes function effectively as drug carriers, enhancing bioavailability and therapeutic outcomes [41,43].

The choice of preparation method depends on factors like drug encapsulation efficiency, stability, scalability, and cost-effectiveness. Thin-film hydration is the most commonly used method since it is simple, suitable for both hydrophilic and hydrophobic drugs, and ensures high drug encapsulation efficiency. However, this method is difficult to scale up due to solvent removal steps, and it requires additional processing (e.g., extrusion, sonication) to control vesicle size. On the other hand, the microfluidic method has the highest potential for industrial scale-up. Controlled mixing of the organic phase, consisting of lipids and bile acids, with the aqueous phase, containing the drug in microfluidic channels, leads to the self-assembly of bilosomes with precise control over vesicle size. This method, however, requires specialized equipment, and optimization is still needed for high-throughput production [24,44,45].

## 5. Administration Routes and Therapeutic Applications

### 5.1. Transdermal Application

Skin delivery represents a promising alternative to oral, parenteral, and inhalatory drug delivery, circumventing their impediments, and thus a highly attractive yet challenging field of scientific research, especially in the last decades. In general, two skin delivery types can be distinguished, namely dermal (topical) and transdermal drug delivery. Topical delivery enables the drug to be directly and rapidly delivered to the site of action on the skin surface, consequently increasing its concentration and local bioavailability. Conversely, transdermal drug delivery systems (TDDSs) can alleviate the permeation of the drug through the skin into the systemic circulation, where therapeutic concentrations are to be achieved. As skin is the largest human organ comprising 15% of the body weight and 2 m^2^ of the body surface, skin delivery possesses significant therapeutic potential. In addition, its remarkable advantages include the avoidance of GI hostility and hepatic first-pass metabolism, controlled drug release and reduced fluctuations of blood concentration, and the ability to rapidly terminate administration. Furthermore, its non-invasive and pain-free nature, comfortable application, and reduced risk of severe adverse effects result in high patient compliance [46,47,48,49]. The attractiveness of topical and transdermal products is also reflected in the value of the Global Cosmetic Skin Care Market, which was estimated at $139.4 billion in 2023 and projected to reach a staggering $197.2 billion by 2030 [50]. Nevertheless, epidermal barriers, and particularly nonviable layer stratum corneum consisting of 15–30 layers of keratinized corneocytes enclosed in a lipid matrix, significantly impede drug absorption and represent a challenging obstacle to overcome. In the attempt to secure therapeutic efficacy and improve the drug’s transdermal permeation, TDDSs have been consistently refined over the past several decades. Nanocarriers have recently emerged as promising representatives of fourth-generation TDDS, due to their abilities to promote drug solubility, transdermal absorption, and bioavailability [46,51]. In the broad range of innovative drug vehicles for transdermal delivery, bilosomes have recently garnered notable scientific attention, whereas their advantageous properties can be chiefly attributed to their distinguished chemical composition (Table 1).

Table 1 presents an overview of a broad spectrum of active substances formulated as bilosomes and investigated for dermal/transdermal application. As previously mentioned, in addition to bile acids, bilosomes consist of lipid matter and non-ionic surfactants. The most commonly utilized lipid components are phosphatidylcholine (PC), lecithin, and cholesterol (CHOL). Phospholipids possess an affinity for biological membranes and increase the hydration of SC which results in lipid structure loosening up and enabling the active component to traverse the skin layer more efficiently and rapidly. Also, they can directly affect the fluidity of the formed vesicles [46,64]. CHOL affects membrane properties, especially in terms of enhancing hydrophobicity and rigidity of the vesicle membrane, increasing its stability, and reducing leakage of the entrapped cargo [73]. Non-ionic surfactants essentially impact the thermodynamic stability of the bilosomal system and increase drug solubility. Furthermore, due to the provided steric hindrance effect, they improve the physical stability of the formulation and positively affect drug absorption. Appropriate HLB value should be selected to improve drug penetration, as it is strongly related to the type of surfactant, noting that both the stratum corneum and the viable epidermis are characterized by lipophilic–hydrophilic gradient [46,49,76]. As presented in Table 1, the most commonly applied non-ionic surfactants are Span^®^, followed by Brij^®^ and Tween^®^. In addition to the types of lipid components and non-ionic surfactants, their amounts and ratios were also varied and investigated. Sodium deoxycholate (SDC) is the most widely investigated bile acid salt in transdermal bilosomal formulations, followed by sodium taurocholate (STC), sodium cholate (SC), sodium glycocholate (SGC), and sodium tauroglycocholate (STCG). The type and amount/concentration of bile salts are varied in order to obtain the optimal formulation.

Thin-lipid film hydration technique is conventionally used to obtain bilosomes. Briefly, a precise amount of active substance, lipid components, and surfactants are dissolved in a suitable organic solvent, which is subsequently evaporated at reduced pressure, resulting in the formation of a dry thin lipid layer. Afterward, the film is hydrated using double-distilled water or phosphate buffer saline containing bile salts. Formed bilosome dispersion undergoes sonication or homogenization to substantially reduce vesicle size and obtain homogeneous distribution [24,69]. With the aim of identifying the variables that might affect the properties of the investigated formulations and obtain the optimized one, factorial designs are widely applied due to their advantageous capability to simultaneously analyze the effect of different variables on the vesicles’ characteristics measured as responses, using the least number of experiments [73,75]. The most commonly investigated dependent variables are entrapment efficiency (EE, %), particle size (PS, nm), polydispersity index (PDI), zeta potential (ZP, mV), and the amount of drug released after a specified period (%) [52,56,57].

As evident in Table 1, Span 60 is the most frequent non-ionic surfactant and SDC bile salt present in the optimized bilosomal formulations. Span 60 is characterized by a longer saturated alkyl chain, lower HLB value than Span 40 and Span 20, and higher phase transition temperature, implying that greater hydrophobicity results in the formation of more stable bilosomes with increased EE. Moreover, the Span:CHOL ratio and the amounts of these components significantly impact the values of the measured responses and formulations’ parameters. Insufficient amount of CHOL in comparison to non-ionic surfactant may significantly decrease the EE due to vesicle instability and drug leakage, whereas an excess of non-ionic surfactant may negatively impact EE due to the formation of mixed micelles and increased solubility of the drug in the dispersion medium. Furthermore, SDC increases the vesicle membrane elasticity and the drug solubility due to its surface-active characteristics, while also preventing the drug leakage due to the lower HLB value, thus positively impacting EE. PS, PDI, and ZP are heavily influenced by the choice of formulation components as well. For instance, the increased surfactant hydrophilicity may result in the expansion of the vesicle’s aqueous core and an increase in the bulk size, whereas high ZP may increase the repulsion force between membrane bilayers, leading to the increase in PS. Bile salts may exert similar effects, due to their anionic nature and steroid structure. It should be noted that PS correlates with the amount of drug entrapped in the nanocarrier. ZP is regarded as a good indicator of the vesicular system’s stability, and ZP values of ±30 mV or higher are desirable, because of the provided strong electrostatic repulsion between the particles of similar charge, which prevents aggregation in the system and improves its stability. A high ratio of Span 60 and CHOL may introduce a negative charge on the bilosomal membrane surface due to the adsorption of the hydroxyl ions and uneven polarity distribution. Similarly, bile acids increase the value of ZP due to their anionic nature. Regarding drug release, bilosomal systems typically show a biphasic release profile, manifested as a rapid initial burst, followed by slower sustained release of the drug, implying that bilosomes act as drug reservoirs. The composition of the nanocarrier significantly impacts the drug release profile, as it determines EE, PS, surface area-to-volume ratio, surface interfacial tension, membrane fluidity and rigidity, and drug solubility, among others. The slow release of the drug may be favorable in the context of transdermal delivery, due to the opportunity to maintain the therapeutic concentrations for a prolonged time period [31,39,52,56,57,59,63]. Furthermore, in vitro and ex vivo permeation studies found higher flux of prepared bilosomes in comparison with their conventional counterparts, which may be attributed to the presence of phospholipids, surfactants, and bile acids, thus reducing particle size, increasing affinity for biological membranes and hydration of stratum corneum, lower viscosity of the formulation, quicker drug release, permeation-enhancing and edge-activating properties of bile acids [39,58,59,60,64,66,67,72,73,74,75].

Moreover, several studies have investigated the possibility of incorporating bilosomal systems into semisolid dosage forms, namely hydrogels, to enhance transdermal drug delivery even further, by providing improved drug solubility, stability, and release profile. Combining the bioadhesive and viscoelastic properties of hydrogels with nanocarriers may provide new solutions in the development of advanced TDDS. Hydrogels absorb water from the surrounding environment, swell, and form 3D network, consequently limiting the access of the dissolution medium and additionally sustaining drug release, which can be acquired in a controlled and steady manner. Furthermore, evident is the use of chitosan as a coating agent as a strategy to improve TDDS. It is reported to offer various benefits, including improved system stability, increased cellular absorption, reduced drug leakage, and enhanced mucosal and skin barrier permeability [31,39,55,56,59,62,65,66,67,69,70,71,72].

### 5.2. Vaccines

The development of vaccines was one of the most significant and major milestones in medical science, resulting in decreased morbidity and mortality, by preventing the spread of devastating diseases and loss of millions of lives. Vaccines are among the most effective public health interventions, with the recent coronavirus pandemic highlighting the global need and urgency for novel and effective vaccination technologies. Despite being of immense benefit in disease prevention, conventional vaccines administered via the parenteral route suffer from several drawbacks, including invasiveness, patient discomfort, high production costs, and the need for well-trained medical professionals. Moreover, although they induce systemic immune response, they do not provide protection against infectious agents at the mucosal level, which is the major port of entry for most of them [77,78,79,80]. Therefore, mucosal immunization emerged as one of the most promising strategies to fight pathogenic microbial infections at the mucosal level of the digestive, respiratory, or urogenital tract [81]. In addition to being advantageous in terms of ease of administration, lower costs of production and greater global accessibility, oral vaccines allow the exposure of gut-based mucosal immune system to antigens and subsequently generate not only heightened localized but also systemic immune response. Furthermore, oral immunization can help induce the production of secretory antibodies at distant mucosal surfaces, due to their connection and communication via the common mucosal immune system [78,79,82]. Nevertheless, the broader application of oral vaccines is greatly challenged by the numerous intrinsic hurdles, including mucosal barriers, a hostile gastrointestinal environment characterized by the wide range of pH values, and the presence of digestive enzymes, bile salts, and the gut microbiome. This necessitates larger and more frequent dosing of the antigen to elicit an adequate immune response, with the subsequent possibility of inducing oral tolerance and systemic non-responsiveness. A variety of nanoparticles have garnered exceptional interest in the realm of vaccine delivery and have been developed and investigated to overcome the aforementioned limitations. By virtue of their unique characteristics, they demonstrate a great potential to achieve efficient oral immunization [78,79,83]. In the context of oral immunization, bilosomes have been in the research spotlight for the past two decades (Table 2).

The potential of structurally modified bilosomes as vehicles for oral vaccine delivery has been studied using bovine serum albumin as a model antigen [84,85]. It was demonstrated that cholera toxin B subunit conjugated bilosomes were successful in increasing both IgG and total intestinal, salivary and vaginal IgA levels in orally immunized female Balb/c mice. Meanwhile, subcutaneous administration of the free antigen produced almost equivalent IgG titer, while failing to provide substantial IgA response. These results may be attributed to the unique composition of the nanocarrier. Cholera toxin B has previously been reported as a mucosal adjuvant that effectively supports antigen specific immune response, while the modified dipalmytoil phosphatidyl ethanolamine served as a long spacer between the adjuvant and bilosomal surface, thus reducing steric hindrance and allowing the interaction of the bilosomes with G_M1_ receptors on M cells [84]. Similar findings were reported using glucomannosylated bilosomes containing bovine serum albumin antigen for oral mucosal immunization. The study revealed improved cellular uptake of the nanocarrier system favored by its vesicular nature and negatively charged surface. Furthermore, extensive uptake by both transcellular and paracellular routes was demonstrated throughout the intestinal region, in comparison with free antigen—a phenomenon that could be ascribed to the small particle size (<200 nm). Immunological studies in male Balb/c mice revealed significantly higher IgG and sIgA levels in male Balb/c mice orally immunized with structurally modified bilosomes, in comparison with alum adsorbed bilosomes and i.m. administered free antigen. These effects may be due to the presence of the protective polymeric coat on the vesicle’s surface, selective receptor-mediated uptake by the major antigen presenting cells in Peyer’s patches, and the possible adjuvant effect of the mannan subunit [85]. Nano-bilosomes were also utilized with the aim of developing oral diphtheria vaccine. Diphtheria toxoid loaded nano-bilosomes successfully stimulated both mucosal and systemic immune responses, although higher doses were required for the oral route in comparison with i.m. administered alum-adsorbed antigen. The bioenvironmental stability of the nano-bilosomes and their efficient uptake in the Peyer’s patches were also confirmed in this study [86]. Several studies investigated the potential of bilosomes as nanocarriers for the oral delivery of influenza antigen [87,88,89,90,91]. Conacher et al. reported that oral vaccination of female BALB/c mice with A. Texas entrapped in bilosomes resulted in the generation of high IgG plasma titers comparable to those obtained by s.c. injection of the same antigen, from week 2 to week 24 post vaccination. Moreover, following oral administration of the entrapped antigen, both Th1 and Th2 immune responses were successfully elicited and production of both serum IgG2a and IgG1 was observed. This finding is of interest, since Th1 associated IgG2a subclass has been associated with the greatest neutralizing capacity against influenza virus [87]. Conversely, Mann et al. were unable to detect any IgG2a production subsequent to oral vaccination with A/Panama antigen loaded bilosomes, although significant IgG1 titers were induced. Additionally, significant mucosal IgA production was reported [88]. However, following studies revealed differential Th1/Th2 bias after immunization with bilosomes of different sizes, due to the differences in the manner in which small (<100 nm) and large (>200 nm) vesicles are trafficked to antigen presenting cells. Larger vesicles were superior in inducing Th1 response and generating higher antibody titers compared with the smaller counterparts, although both alternatives were successful in initiating IgA response [89]. Nevertheless, vaccination with the oral formulation containing large bilosomes resulted in better resistance to infection challenged in vivo, and they were more successful in promoting antigen uptake and retention within the Payer’s patch and mesentery lymph nodes [89,91]. Similar findings regarding the skewing of the immune response toward IgG1 impacted by the smaller vesicle size and a mucosal IgA response were reported by Bennet et al. Interestingly, the same authors reported the immunogenic efficacy of the lyophilized formulations, which may be of great interest due to the improved storage and distribution conditions [90]. Bilosomes were also investigated in the context of immunization against hepatitis B. Subsequent to oral administration, HBsAg loaded bilosomes were successful in inducing the serum anti-HBsAg IgG titers comparable to those obtained after the i.m. application of the alum adsorbed antigen, as well as mucosal antibody response, which was not detected in the case of i.m. vaccination [92].

Furthermore, coupling the modified cholera toxin B subunit to the bilosomal surface led to the effective targeting of the loaded antigen to the M-cells of the GALT and enhanced immunogenicity, allowing for the use of lower doses [93]. Promising findings have been reported for the use of oral bilosome vaccines against *Clostridium tetani* infection. The oral vaccine containing tetanus toxoid (200 μg/mouse) was successful in stimulating the production of IgG1 titers comparable to those obtained by s.c. Application of the same antigen (50 μg/mouse), while no regimen induced IgG2. However, only after oral immunization was there an increase in IgA-positive plasma cells implying efficient needle-free mucosal immunization [94]. Surface-engineered bilosomes were also applied to provide increased stability in GIT and selective uptake and delivery of tetanus toxoid to antigen-presenting cells via the oral route. Mannosylated bilosomes were successful in eliciting good systemic, mucosal, and cellular immune responses [95]. These findings were also corroborated by the study of Gebril et al., who found that bilosome-loaded antigens produced a systemic and local immune response, which was efficient in protecting the mice against toxin challenge [96].

### 5.3. Oral Application

Oral administration remains the most widespread and preferred method of drug consumption, being advantageous in terms of patient preferences, ease of non-invasive self-administration, cost-effectiveness, and large-scale manufacturing, as well as addressing both systemic and local GIT diseases. It has been reported that oral dosage forms represent around 90% of the global market share of all pharmaceutical formulations intended for human use. Nevertheless, oral drug delivery is severely challenged by a number of barriers, including anatomy, biochemistry, and (patho)physiology factors. In addition, the inherent physicochemical properties of drug candidates can substantially reduce and limit their bioavailability and compromise therapeutic outcomes. In this sense, the oral delivery of drugs characterized by low solubility (BCS class II), low permeability (BSC class III), or both (BCS class IV), as well as peptide- and protein-based drugs is particularly challenging. Considering the fact that newer chemical entities and potential drug candidates usually suffer from inadequate solubility–permeability characteristics that impede their bioavailability after oral application, and the great promise that protein therapeutics hold, considerable interdisciplinary efforts have been put into overcoming the aforementioned issues. Orally administered nanocarriers have attracted significant interest, being one of the most promising and innovative strategies to improve tolerability, pharmacological specificity, biodegradability, and targeting of oral drugs [97,98,99,100,101]. Over the years, bilosomes have garnered significant attention for the optimized oral delivery of various natural, novel, and well-established therapeutic compounds for the treatment of a broad spectrum of diseases (Table 3).

Bilosomes were investigated as nanocarriers for the oral delivery of antibiotic phytochemical lycopene and well-established antimicrobials levofloxacin and doxycycline. In vitro and in vivo potential of lycopene against *K. pneumoniae* isolates was potentiated by bilosome encapsulation [103], while levofloxacin and doxycycline-loaded bilosomal formulations emerged as significantly more efficacious in increasing survival rates of *Burkholderia*-infected mice than free drugs. Interestingly, no hazardous effects on the microbiome were recorded, implying the potential of bilosome encapsulation as a strategy to minimize serious GIT side effects [104]. The same strategy coupled with freeze drying provided a notable sustained release for up to 24 h to BCS class II anti-depressant sertraline, and a 222% bioavailability enhancement in an in vivo absorption study in rats, compared to the free drug [105]. Several phytochemicals were scrutinized for the enhanced anti-diabetic potential subsequent to bilosome entrapment. Pharmacokinetic studies showed increased systemic bioavailability resulting in the boosted hypoglycemic activity of the apigenin and berberine-loaded bilosomes [106,107]. Lactoferrin-coated bilosomes encapsulating quercetin exhibited superior antidiabetic effects relative to both the uncoated counterpart and a free molecule, at the biochemical, cellular, and molecular levels, implying the synergy between their hypoglycemic, anti-inflammatory, and antioxidant effects [110]. Over the years, tremendous scientific effort has been directed toward enabling the oral delivery of insulin, a staple antidiabetic of vital importance for over 500 million adults living with diabetes. Bilosomal formulations have been reported to improve insulin intestinal absorption [108]. Moreover, anionic bilosomes demonstrated higher cellular uptake using Caco-2 cell lines compared to their cationic counterparts, indicating the contribution of ASBT uptake, whereas in vivo animal studies revealed a 1.6-fold increase in AUC achieved by the oral insulin-loaded bilosomal formulation, compared to s.c. insulin injection [109]. Promising results were published for eprosartan mesylate-loaded bilosomes, which successfully exhibited renoprotective effects in the animal diabetes nephropathy model [111]. A plethora of natural products are characterized by well-documented antioxidant, anti-inflammatory, antibiotic, and cytotoxic potential which makes them highly promising therapeutic alternatives. However, their broader application is severely impeded by the unfavorable physicochemical properties regarding solubility and permeability. Bilosomes were utilized as an efficient strategy to improve the oral delivery of several phytochemicals for the management of different diseases, improving their GIT stability and bioavailability while prolonging the release time, rendering them the capacity to fully exhibit their therapeutic potential [77,114,115,116,117,118]. Additionally, surface modification of the nanocarrier with polyethylene glycol 2000 [117] and chitosan [114,118] augmented the cytotoxic and antibacterial activity of the loaded phytochemicals, by various mechanisms which include mucoadhesion, sustained release, and inherent antibacterial activity of the applied additive. Bile salt-containing vesicles were also investigated as carriers for improved oral delivery of various antiviral agents. Acyclovir suffers from low bioavailability and a short half-life. However, its relative bioavailability was substantially improved by entrapment into bilosomes and was 2.5-fold higher than the value obtained for the tested marketed product [119]. The galactose-anchored taurocholate-containing bilosomes were efficient in increasing sofosbuvir hepatic availability, due to the enhanced drug absorption and successful liver targeting [121]. In vitro studies suggested 6.6 times improvement in the antiviral activity of bilosome-entrapped resveratrol in comparison with phytochemical dispersion, while piperine-containing bilosomes demonstrated superior antiviral, anti-inflammatory, and antioxidant activity over piperine suspension, highlighting their potential as prophylactic or therapeutic agents against SARS-CoV-2 and MERS-CoV [120,122]. Natural products are also garnering significant attention as promising cytotoxic agents, while, at the same time, targeted delivery of conventional antineoplastic drugs remains a challenge in carcinoma treatment. Nanotechnology has been widely employed to overcome the existing impediments to successful chemotherapy. Surface engineering was utilized as a strategy to improve drug targeting or accentuate the cytotoxic effects of several anti-cancer candidates formulated as bilosomes. In vitro studies revealed that D-alpha-tocopheryl polyethylene glycol succinate decorated curcumin-containing bilosomes demonstrated higher cytotoxicity against doxorubicin-resistant breast cancer cell lines, by the virtue of attenuating P-gp efflux pump [123]. Melittin was selected as a component of icariin-loaded bilosomes to additionally potentiate its cytotoxic and pro-apoptotic activities [124]. A similar effect was reported for piceatannol-entrapped bilosomes containing zein, which potentially contributed to the overall improvement of the cytotoxic potential against lung cancer cells [125]. Lactoferrin was utilized as a coating agent for pitavastatin-loaded bilosomes to facilitate receptor-mediated endocytosis over-expressed in hepatocellular carcinoma, and the optimized formulation exhibited highly favorable anticancer potency, both in vitro and in vivo [126]. Chitosan-decorated bilosomes loaded with psoralidin hold promise in addressing breast and lung cancers, due to the enhanced apoptotic and necrotic effects [127]. Dextrose-functionalized silymarin bilosomes for liver-specific peroral delivery were reported as potent chemotherapeutic agents against hepatic carcinoma, due to the improved localization and internalization of the therapeutic agent into tumor cells [129].

The potential of bilosomes as finely tunable nanocarriers for the improved oral delivery and bioavailability of various therapeutic agents lies in their specific composition, which grants them several advantageous properties. Successful entrapment of the drug in secured by the strategically selected lipid components and surfactants, which may act as solubilizing agents, penetration enhancers, and P-gp efflux pump blockers. Additionally, bile acids provide vesicles with elasticity, enhance their permeability, and protect their integrity in the hostile GIT microenvironment. Vesicle nanosize provides a significantly higher surface area which facilitates the permeation process. Also, a negative particle charge promotes drug uptake by M-cells of Peyer’s patches and enhances its absorption through the intestinal lymphatic transport pathway, which helps evade extensive first-pass metabolism in the liver. Overall, bilosome encapsulation represents an effective strategy to overcome several critical impediments to efficient oral drug delivery, including insufficient drug absorption, low bioavailability, multiple-dose regime, and associated side effects [105,119,123].

### 5.4. Intranasal Administration

In the past century, intranasal drug administration was predominantly limited to treating topical symptoms of various respiratory diseases. However, unraveling the possibility of drug conveyance to the central nervous system (CNS) via nasal pathways by the end of the 20th century gave rise to the rapidly developing area of research regarding nose-to-brain delivery. Interestingly, around threefold increase in the number of interventional intranasal studies was observed in the past decade, compared with the period from 2000 to 2010. The attractiveness of intranasal drug delivery lies in its several advantages over conventional routes of drug administration, including non-invasive and easy access, rapid drug absorption and onset of action, as well as the annulment of the first-pass drug metabolism. Of particular interest is the remarkable possibility to bypass the blood–brain barrier and provide direct access to the brain via olfactory and trigeminal pathways. Thus, the intranasal route of drug administration holds great potential for the treatment of CNS disorders. However, poor permeability of nasal mucosa to large hydrophilic or highly charged drug molecules, variable bioavailability, prompt mucociliary clearance, enzymatic degradation, and insufficient drug retention time significantly impair and obstruct therapeutic efficacy [136,137,138,139]. Nevertheless, recent advancements in nanotechnology show promise in overcoming current intranasal drug delivery limitations. Among the studied nanocarriers, bilosomes have recently garnered research attention (Table 4).

In the search for novel treatments for Alzheimer’s disease (AD), SDC-containing bilosomes encapsulating poorly bioavailable phytochemicals luteolin and resveratrol for intranasal application have been investigated [140,141]. Optimized and intranasally applied luteolin-loaded bilosomes demonstrated superiority over luteolin suspension in improving short-term and long-term spatial memory in mice with streptozotocin-induced AD. Moreover, the bilosomal formulation enhanced the antioxidant, anti-inflammatory, and anti-amyloidogenic properties of luteolin, highlighting its therapeutic potential for managing AD [140]. Abbas et al. investigated the effects of resveratrol-loaded chitosan-coated bilosomes that were additionally incorporated into sodium alginate/PVP 90 wafers in a lipopolysaccharide-induced AD animal model. Moreover, superparamagnetic iron oxide nanoparticles (SPIONs) were loaded into chitosan-coated bilosomes to target the brain by the use of an external magnetic field. Bilosomes provided high entrapment efficiency, high dispersion homogeneity, and small particle size. Wafers form in situ gel when in contact with mucosa, which is greatly beneficial in terms of extending drug residence time, sustaining drug release, loading poorly water-soluble APIs, and ultimately improving drug absorption and therapeutic effects. In vivo studies showed that nanocarrier-loaded wafers were superior over resveratrol suspension and allowed for efficient delivery of the phytochemical to the brain via the olfactory mucosa, which was manifested by improved memory and cognitive functions in mice with induced AD. These findings can be attributed to the nanoencapsulation of resveratrol and its improved solubility and bioavailability, prolonged residence time at the application site, and enhanced absorption, additionally enhanced by the wafer effects. Of interest, SPION-loaded bilosomes could be guided to the brain even more efficiently by applying an external magnetic field [141]. Doxylamine succinate and pyridoxine hydrochloride loaded bilosomes were fabricated as thermally-triggered in situ poloxamer 407:poloxamer188:Carbopol 971P gels with the aim of addressing pregnancy-allied nausea and vomiting. The obtained gel system provided quick absorption, improved bioavailability, and prolonged residence time, in comparison with both the intranasal-free in situ gel and oral solution. The observed effects were due to the augmented permeability, fluidity, and high surface area provided by the bilosomes, as well as the extensive mucoadhesive properties of the incorporated polymers [142]. A similar strategy was applied to deliver intranasal zolmitriptan directly to the brain, through the fabrication of bilosomes incorporated into a mucoadhesive in situ gel consisting of HPMC and poloxamer 407. Pharmacokinetic studies revealed that intranasal application of both bilosomal dispersion and in situ gel resulted in brain targeting, while the relative brain bioavailability of the zolmitriptan from in situ gel was 1.4-fold greater than that from dispersion. In addition, the olfactory pathway was identified as the dominant delivery route for zolmitriptan to reach the brain. Successful nose-to-brain delivery can be ascribed to the small particle size and elasticity of bilosomes, facilitating both intracellular and extracellular drug transport to the brain, which is additionally enhanced by the mucoadhesive properties of the gel system enabling better drug residence, absorption, and penetration [144]. The anti-inflammatory efficacy of budesonide entrapped in SC-containing bilosomes against the market product was investigated in potassium dichromate-induced inflammation and lung injury in rats. Bilosomal formulation reduced systemic exposure and prolonged the drug’s release at the desired site, effectively targeting lung tissue and significantly decreasing inflammation, thus representing a promising platform to enhance and maximize the therapeutic efficacy of the aforementioned corticosteroid [142].

### 5.5. Ocular Application

Successful ocular drug delivery has remained a challenge in ophthalmic disease treatment for decades. Nevertheless, worldwide scientific interest in ocular drug delivery has been steadily increasing over the past two decades, making this interdisciplinary field highly attractive and rapidly developing. Although many potent ophthalmic drugs are available and accessible, the anatomic complexity of the eye and numerous precorneal, corneal, and blood–ocular barriers, in addition to formulation constraints, significantly hinder their therapeutic efficacy. In fact, due to the aforementioned impediments, the bioavailability of the topically administered drugs can be reduced to less than 5%. Recent bibliometric analysis has revealed that nanosystems represent a current trend and focus of the research interest in the domain of ocular pharmaceutical science since they are emerging promising and innovative solutions to overcome ocular barriers, prolong drug retention time, increase its bioavailability, and enhance treatment efficacy [145,146,147,148]. In recent years, bilosomes have gained attention as potential nanocarriers for several antimicrobials and glaucoma medications for improved ocular pharmacotherapy (Table 5).

To ensure adequate drug bioavailability, it is of vital importance to improve and extend the residence time of the drug in the pre-corneal space. Various formulations such as ointments, contact lenses, ocular inserts, and collagen shields have been reported to provide the desired effect, however, they suffer from several drawbacks, including difficulty in administration, blurred vision, or patient noncompliance, which is of pivotal concern. In situ hydrogels, which exist in sol form under normal conditions and undergo a phase transition to gel in response to adequate stimuli (pH, electrolytes, temperature), show better patient compliance. This approach was applied to formulate ciprofloxacin, moxifloxacin, and natamycin-loaded bilosomes in situ gels for ocular delivery [149,150,151]. Carbopol 934P and HPMC 100 K were utilized to formulate ocular hydrogel containing optimized ciprofloxacin-loaded bilosomes. The optimized bilosome containing hydrogel exhibited suitable gelling strength, viscosity, bio-adhesiveness, and drug content. In vitro release studies revealed sustained release of ciprofloxacin from the optimized bilosomes, which was even further prolonged by incorporating bilosomes into an in situ gel system. Ex vivo trans corneal permeation studies confirmed significantly higher permeation of ciprofloxacin when incorporated into bilosomal hydrogel, with the enhancement ratio being 3.08 higher than pure ciprofloxacin. The aforementioned effects can be attributed to the nano size of the formulation and its components, which may interact with the tear film, enhance corneal permeability, and prolong ocular residence time. Of interest, safety studies and a greater antimicrobial potential of the novel formulation corroborate its viability as a carrier for improving the therapeutic efficacy of the conventional antibiotic [149]. Similar results were obtained for moxifloxacin-loaded bilosomes in situ gel containing sodium alginate and chitosan. Optimized ocular hydrogel demonstrated a 2.8-fold increase in permeation of moxifloxacin than the pure solution, due to the components’ interaction with the tear film, increased residence time, and reduced drug loss by tear fluid turnover, as well as significantly higher antimicrobial activity and no irritation potential [150]. Gellan gum/xanthan gum in situ gel comprising natamycin-loaded bilosomes provided superior permeability to the drug in comparison to control suspension, emerging as another potential nanocarrier system for improved and extended topical ocular antibiotic delivery [151]. Edge activators can be employed to additionally increase the permeation of drug-loaded bilosomes. Ex vivo corneal permeation study revealed the superiority of the optimized STC and Cremophor EL containing bilosomes encapsulating terconazole over conventional bilosomes, niosomes, and suspension of the same antifungal agents, in all investigated permeation parameters. In addition to the lipid fluidizing effect of the bile salts, such findings can be explained by the higher elasticity of the edge activator containing nanocarriers, which permits them to squeeze through the cornea and pass through the aqueous humor into vitreous without rapturing. Importantly, in vivo ocular tolerance tests and histopathological studies confirmed the non-irritant properties of the applied ultradeformable bilosomes in animals [152]. Self-nanoemulsifying system containing optimized terconazole-loaded bilosomes emerged as yet another auspicious strategy to improve antifungal’s therapeutic efficacy. The aforementioned integrated dual system provided an immediate release of the drug by the virtue of self-nanoemulsifying component, followed by sustained release offered by bilosomes, longer residence time, and enhanced antifungal activity against *C. albicans*-induced fungal keratitis in the animal model [153].

Bilosomes have been employed and investigated as potential nanocarriers for several antiglaucoma drugs [154,155,156,157]. In vivo studies revealed that agomelatine-loaded bilosomes comprised of SC and hyaluronic acid achieved much higher intraocular pressure decrease than that obtained by the drug solution (82.682% vs. 35.92%), in addition to prolonging drug mean residence time. Thus, the higher bioavailability of the optimized bilosomal formula could be ascribed to its penetration-enhancing components (PC and SC) and hyaluronic acid which prevents rapid drug clearance [155]. Ultradeformable bilosomes comprising edge activators (Cremophor EL, Tween 80) were fabricated to enhance the corneal permeation of betaxolol hydrochloride and brinzolamide. This approach proved to be fruitful in increasing the pliability and malleability of the nanovesicles, which resulted in alleviated penetration through the cornea and efficient transport of the loaded cargo [156,157]. Similar results were obtained for acetazolamide-loaded bilosomes, which helped achieve an improved pharmacodynamic profile and enhanced ocular bioavailability in comparison with niosomes, marketed dorzolamide eye drops, and marketed acetazolamide tablets [154].

### 5.6. Buccal Application

The buccal route of drug administration represents an attractive and extensively studied alternative to oral and intravenous drug delivery. Buccal mucosa is non-keratinized elastic and permeable tissue located on the surface of the inner cheek, which makes it highly accessible, and well-suited for the systemic delivery of therapeutic agents. In addition, it is advantageous in terms of circumventing the hepatic first-pass metabolism, low enzymatic activity, and mitigating potential side effects. In 2021, the global buccal drug delivery market was estimated at $3.2 billion, and projected to reach $7.13 billion by 2030. Nevertheless, the relatively small surface area (50 cm^2^), low permeability, and the continuous secretion of saliva leading to drug loss represent the most prominent limitations to buccal drug delivery [158,159,160]. To address the aforementioned issues, the nanocarriers and mucoadhesive (bio)polymers have been widely investigated and represent promising strategies for the successful delivery of drugs via buccal mucosa [161]. So far, several drugs have been incorporated into bilosomes and tested for buccal drug delivery (Table 6).

Alternatives to the subcutaneous route of insulin delivery have been garnering scientific attention for years. Elastic bilosomes incorporating insulin and containing different bile salts have been investigated in the in vitro TR146 cell culture model. Fabricated nanocarriers enhanced the insulin permeability across TR146 cells in comparison with insulin solution, due to their ability to deform and squeeze through the cell pores, as well as the penetration-enhancing effects of the chosen bile salts [162]. Carvedilol and vardenafil are both classified as a class II in the BCS, due to their high permeability and low water solubility. Both of the aforementioned therapeutics have been loaded into SDC-containing bilosomes and further incorporated into mucoadhesive nanosponges, with the aim of utilizing the advantages of both platforms and enhancing drugs’ bioavailability [163,164]. Mucoadhesive drug delivery systems offer advantages in terms of prolonged residence time and more efficient absorption, leading to a lower frequency of drug administration, reduced dose-related side effects, and improved bioavailability [158,165]. Cellulose derivatives have been applied in the development of carvedilol and vardenafil bilosomal mucoadhesive nanosponges. Ex vivo permeation studies showed that the transdermal flux of carvedilol was significantly increased from optimized bilosomal formulation compared with carvedilol aqueous suspension. This effect may be attributed to the small vesicular size, as well as the presence of phospholipids and SDC, providing the affinity to the biological membranes and opening their tight junctions, thus facilitating drug permeation. In addition, carvedilol bilosomal sponge was characterized by the swelling ratio adequate for buccal delivery, relatively long mucoadhesion time, and prolonged in vitro drug release. In vivo studies demonstrated its superior ability to efficiently reduce both systolic and diastolic blood pressure to normal control levels in CdCl_2_-induced hypertension in rats and exhibit cardio-protective effects, compared to the commercially available product [163]. Similar findings have been reported for vardenafil-loaded bilosomal nanosponges, which exhibited appropriate mucoadhesion time and efficacy in enhancing the ex vivo permeation of vardenafil. In vivo studies demonstrated that vardenafil-loaded bilosomal nanosponge for buccal application efficiently enhanced the drug’s pharmacokinetics and cGMP serum levels, in comparison with vardenafil suspension, highlighting its superior in vivo therapeutic potential [164].

### 5.7. Vaginal Application

The interest in developing vaginal drug delivery systems has been remarkably increasing in recent decades, owing to their notable advantages over conventional dosage forms, whose effectiveness is majorly limited by the present biological barriers. The rise of nanotechnology allowed for various nanocarriers to be fabricated and tested for vaginal drug delivery, including liposomes and their structural derivatives [166,167].

Albash et al. prepared and characterized novel ultra-deformable liposomes containing phosphatidylcholine, terpenes, SDC, and ethanol with the aim of enhancing permeation of fenticonazole nitrate through the vaginal mucous membrane. Box-Behnken design was applied to obtain the optimal formulation (Table 7). Ex vivo permeation studies showed that the optimized formulation was characterized by a higher enhancement ratio than classical bilosomes (3.65-fold vs. 2.28-fold), while both nanocarriers were superior to drug suspension. In addition, nanocarriers provided for a higher amount of drug to be deposited in vaginal tissue. Optimal formulation incorporated into HMPC-based intravaginal gel exhibited significant antifungal potential against *C. albicans* in animal vaginal candidiasis model, as well as safety confirmed by histopathological studies. The aforementioned effects may be corroborated by the optimized size of the nanocarriers, as well as their deformability potentiated by the terpenes, ethanol, and SDC [168].

## 6. Limitations of the Use of Bilosomes

Despite many advantages of bilosomes, such as better stability in comparison to liposomes and some other vesicular drug delivery systems, and better improvement of drug bioavailability after peroral use, but also their superiority over other administration routes, they possess several limitations as well. Although usually consisting of safe natural components (phospholipids, bile salts), some other surfactants in bilosome formulation may cause cell toxicity or irritation. While bile salts are generally recognized as safe, they can also exert some adverse effects; however, most of these side effects depend on factors such as the type and concentration of bile salts used, the specific drug being delivered, and individual sensitivity. While considered generally stable, bilosomes still may face physical and chemical instability, such as aggregation, leakage of the encapsulated drug, or degradation over time. The preparation of bilosomes involves multiple steps, requiring precise optimization of formulation parameters; differences in size, charge, and drug loading may arise during production, affecting reproducibility. As previously stated, industrial-scale production and regulatory approval are currently one of the major concerns due to the complexity of formulation and preparation methods, as well as the lack of standardized testing methodologies and criteria.

## 7. Concluding Remarks and Future Directions

Bilosomes are versatile drug delivery systems that improve the bioavailability of therapeutic agents, as well as their stability. Their unique lipid-based vesicular structure makes them suitable for administration via various routes, including peroral, transdermal, buccal, nasal, ocular, vaginal, and parenteral. They are particularly effective as vaccine delivery systems due to their ability to encapsulate antigens, protecting them from degradation and enhancing their stability, as well as stimulating both humoral and cellular immunity following the non-invasive vaccine administration routes. By improving drug absorption and minimizing side effects, bilosomes offer significant potential for efficient and patient-friendly therapies. These advantages may also be beneficial in improving the outcomes of anticancer and antibiotic treatment, as well as in enhancing the therapeutic potential of various phytochemicals.

Considering the previously described drawbacks that limit the use of bilosomes, future research directions are mostly oriented toward developing new cost-effective, reproducible, and scalable production methods. These approaches should prioritize green and sustainable practices, to ensure the long-term viability of large-scale production and reduce environmental impact. The functionalization with ligands (e.g., antibodies) for improved site-specific drug delivery is also in the research focus. In the era of personalized medicine, tailoring bilosome formulations based on patient-specific factors would be of great significance, along with AI-driven optimization of bilosome properties for individualized treatments. Consequently, future research must critically address the safety aspects of both acute and long-term bilosome applications, focusing on potential immunogenicity, toxicity, and the risks of chronic exposure. Comprehensive studies should investigate the biocompatibility, stability, and degradation of bilosome formulations, ensuring that their use does not result in harmful cumulative effects or unforeseen adverse reactions. Such research is essential for ensuring the overall safety and efficacy of bilosome-based therapies in clinical settings.

## Figures and Tables

**Table 1:** Summary of studies investigating the transdermal use of bilosomes.

Active Substance	Investigated Activity ^a^	Bilosome Composition	Formation Technique/Optimization	Optimal Formulation	Irritability Test	Ref.
Dapsone	antiacne	Span 60:CHOL (5:1, 10:1 molar ratio), SDC, SC, STC (0.25–0.5 M)	Thin-film hydration/ 3^1^∙2^2^ full factorial design	Span 60:CHOL 5:1 molar ratio, SDC 0.25 M	Histopathological study (safe)	[52]
Luteolin	antiaging	L-α-PC:CHOL 4:1 weight ratio, SDC (0%, 10%, 25% *w*/*w* of PC)	Thin-film hydration	/	Draize test (score < 2), histopathological study (safe)	[53]
*Spirulina platensis*	antiaging	L-α-PC, CHOL, SDC (10 mg, 25 mg)	Thin-film hydration	/	Draize test (score < 2), histopathological study (safe)	[54]
Dronedarone	antiarrhythmic	Span 40:CHOL (1:1 molar ratio), SDC (0.2% *w*/*v*), clove oil (1.5–3% *w*/*v*), Tween 60 (25–50 mg), Tween 80 (25–50 mg), NaCMC (1% *w*/*v*)	Ethanol injection/ 2^3^ full factorial design	Tween 60 (50 mg), clove oil (3% *w*/*v*)	pH within the non-irritating range	[55]
Empagliflozin	antidiabetic	Span 40, Span 60, CHOL (Span:CHOL mixture 100–300 mg, Span:CHOL 3–9 ratio) SDC, STGC (5–25 mg), Carbopol 940 (0.5–1%), Carbopol 934 (1–1.5%), HPMC (5%), NaCMC (5%)	Modified injection method/I-optimal design	Span 60:CHOL 9:1 mixture 139.59 mg, STGC 25 mg, Carbopol 940 1% (gel:bilosome 1:1)	Histopathological study (safe)	[56]
Metformin hydrochloride	antidiabetic	CHOL, Span 40, Span 60, SC, SDC, STC (8 mg, 14 mg)	Solvent evaporation/ 3^1^∙2^2^ full factorial design	Span 60, SDC 8 mg	/	[57]
Ondansetron hydrochloride	antiemetic	Span 60, Span 80, CHOL (Span:CHOL 7:0, 7:1, 7:3 molar ratio), SDC (0–5% *w*/*v*)	Thin-film hydration/ 3^2^∙2^1^ full factorial design	Span 60:CHOL 7:1, SDC 2.5% *w*/*v*	In vivo histopathological study (safe)	[58]
Butenafine	antifungal	PC (1.5–4.5%), Span 60 (30–60 mg) CHOL, SDC (15–25 mg), Carbopol 940 (1% *w*/*v*)	Thin-film hydration/ Box-Behnken design	PC 3.5%, Span 60 45 mg, SDC 22 mg	HET-CAM test (score 0)	[59]
Miconazole nitrate	antifungal	CHOL (20 mg, 40 mg), Tween 80 (2%, 4%), SDC (20 mg, 30 mg), Carbopol 934P (0.5–1% *w*/*v*), Chitosan (0.5% *w*/*v*)	Thin-film hydration/ Box-Behnken 3^3^ statistical design (BBD)	CHOL 30 mg, Tween 80 4%, SDC 30 mg, Carbopol 934P 1%	HET-CAM (score 0)	[39]
Terconazole	antifungal	CHOL (25 mg), Span 60 (100 mg), SDC, SGC (5–15 mg), Brij-93, Cremophor EL (5 mg)	Thin-film hydration/ 2^4^ complete factorial design	CHOL 25 mg, Span 60 100 mg, SDC 5 mg, Brij 93 5 mg	Histopathological study (safe)	[60]
Olmesartan medoxomil	antihypertensive	L-α-PC, Cholesterol (25 mg), Span 60 (50 mg), SDC (5 mg), STC (15 mg), Brijo20 (15–25 mg), Brij 52 (15–25 mg)	Thin-film hydration/ 2^4^ full factorial design	STC (15 mg), Brij 52 (15 mg)	In vivo histopathological study (normal)	[61]
Sildenafil citrate	antihypertensive	SPC (200–400 mg), CHOL (20 mg), Span 60 (40–60 mg), SDC (10–30 mg), HPMC (2% *w*/*w*)	Thin-film hydration/ 3^3^ Box-Behnken design	SPC 251.5 mg, SDC 30 mg, Span 60 60 mg	/	[62]
Valsartan	antihypertensive	Span 20, Span 40, Span 60, CHOL (Span:CHOL 1:1, 8:2, 2:8), SDC (5–20 mg), Carbopol 940 (1% *w*/*v*)	Thin-film hydration/ 3^3^ full factorial design	Span 60:CHOL 8:2, SDC 20 mg	HET-CAM test (score 0), histopathological study (safe)	[63]
Berberine chloride	anti-inflammatory	Soybean lecithin (2.5–5% *w*/*v*), CHOL, SDC (5–15% *w*/*v*), Chitosan (0–0.25% *w*/*v*), Carbopol 974 NF (2%)	Thin-film hydration/ 3^3^ Box-Behnken design	Soybean lecithin 5%, SDC 5%, Chitosan 0.16%	Histopathological study (safe)	[64]
Curcumin	anti-inflammatory	CHOL, Span 60 (CHOL:Span 1:5, 1:10 molar ratio), SC, SDC (5 mg, 10 mg), Alginate dialdehyde/Chitosan cross-linked hydrogel	Thin-film hydration/ 2^3^ factorial design	CHOL:Span 60 1:10, SDC 5 mg	Histopathological study (regenerative potential)	[65]
Terbutaline sulfate	bronchodilator	Soybean PC (3–5% *w*/*v*), CHOL (20 mg), SDC (5–15% *w*/*v*), Chitosan (0–0.3% *w*/*v*), HPMC K15M (2% *w*/*w*)	Thin-film hydration/ Face-centered central composite design, artificial neural network modeling	Lipid 5% *w*/*v*, SDC 8% *w*/*v*, Chitosan 0.06%	In vivo histopathological study (safe)	[66]
Fluticasone propionate	corticosteroid	PC90G (1–3%), SDC (0.02–0.1%), STC (0–0.1%), stearylamine (0–0.3%), Carbopol 940 1% (*w*/*w*)	Thin-film evaporation/ 4^3^ Draper-Lin small composite design	PC90G 2.99%, SDC 0.04%, stearylamine 0.29%	Histopathological study (regenerative potential)	[67]
Tamoxifen	cytostatic	PL90G (2–4%), STC or SC (0.2–0.4%), ethanol (15–25%)	Cold method with ethanol and extrusion/ 2^4^ full factorial design	PL90G 2%, STC 0.4%, ethanol 25%; PL90G 4%, SC 0.2%, ethanol 25%	/	[68]
Simvastatin	hypolipidemic	Soy PC (1–3 molar concentration) CHOL, Span 60 (30–60 mg), SDC (10–30 mg), HPMC (2% *w*/*w*)	Thin-film hydration/ Box-Behnken 3^3^ statistical design (BBD)	Lipid 1.427%, Span 60 60 mg, SDC 30 mg	/	[69]
Tizanidine hydrochloride	myorelaxant	Span 20, Span 40, Span 60, CHOL (Span:,CHOL 3:7, 1:1, 7:3 molar ratio), SDC (5–20 mg), CMC (5% *w*/*w*)	Thin-film hydration/ 3^3^ full factorial design	Span 60:CHOL 1:1 molar ratio, SDC 20 mg	/	[70]
Diclofenac sodium	non-steroidal anti-inflammatory drug	Lecithin, CHOL, Span 40, Span 60, SC, SDC, SGC (8 mg, 18 mg), Carbopol 971P (2% *w*/*w*)	Thin-film hydration/ 3^1^∙2^2^ full factorial design	Span 60, SDC 18 mg	Histopathological study (normal periarticular and soft tissues)	[71]
Lornoxicam	non-steroidal anti-inflammatory drug	Soybean PC (0.02–0.06 M), SDC (10–30 mg), limonene (0.25–0.75%), Carbopol (1% *w*/*w*)	Thin-film hydration/Box-Behnken 3^3^ full factorial design	Lipid 0.02 M, SDC 10 mg, limonene 0.47%	Draize test (score 0), histopathological study (safe)	[31]
Niflumic acid	non-steroidal anti-inflammatory drug	Brij-93, Brij-35 (5 mg), Span 20 (100 mg), CHOL (25 mg), SC, STC, SGC (5 mg, 15 mg), HPMC (2%)	Ethanol injection/3^1^∙2^2^ full factorial design	Brij-93, STC 5 mg	Histopathological study (regenerative potential)	[72]
Tenoxicam	non-steroidal anti-inflammatory drug	Span 40, Span 60, Span 80, CHOL (Span:CHOL 5:1, 5:3 molar ratio), SDC (0.5 M, 0.25 M)	Thin-film hydration/ 3^1^∙2^2^ full factorial design	Span 60:CHOL 5:1 molar ratio, SDC 0.25 M	In vivo histopathological study (no obvious skin irritation)	[73]
Sildenafil citrate	PDE-5 inhibitor	Pure soybean PC (3% *w*/*v*), SDC, DCA, STGC (soybean PC:BA 4:1, 6:1, 8:1)	Thin-film hydration and Ethanol injection method/3^3^ Box-Behnken design	Pure soybean PC:STGC 4:1	Skin integrity test (no skin defects)	[74]
Diacerein	structural modifying osteoarthritic drug	CHOL, Span 40, Span 60, SC, SGC, STC (5 mg, 15 mg)	Thin-film hydration/ 3^1^∙2^2^ full factorial design	Span 60, STC 15 mg	In vivo histopathological study (safe)	[75]

^a^ In alphabetical order. Abbreviations: PC, phosphatidylcholine; BA, bile acid; CHOL, cholesterol; DCA, deoxycholic acid; SC, sodium cholate; SDC, sodium deoxycholate; SGC, sodium glycocholate; STC, sodium taurocholate; STGC, sodium tauroglycocholate; CMC, carboxymethylcellulose; NaCMC, sodium carboxymethyl cellulose; HPMC, hydroxypropyl methylcellulose.

**Table 2:** Summary of studies investigating the use of bilosomes in vaccines.

Active Substance	Investigated Activity	Bilosome Composition	Formation Technique/Optimization	Optimal Formulation	Ref.
Bovine serum albumin	vaccine	Sorbitan tristearate:CHOL:modified dipalmytoil phosphatidyl ethanolamine (7:3:1), SDC (20 mg), modified cholera toxin B subunit	Thin-film hydration	/	[84]
Bovine serum albumin	vaccine	Span 80:CHOL:SDC (2:1:0.1–0.4 molar ratio), stearylamine, GM-OCM-DSPE (5–15% *w*/*w*)	Thin-film hydration	Span 80:CHOL:SDC 2:1:0.1 molar ratio, GM-OCM-DSPE 10%	[85]
Diphtheria toxoid	vaccine	Sorbitan tristearatae:CHOL:diacetyl phosphate (7:3:1 molar ratio), SDC (100 mg)	Thin-film hydration	/	[86]
Synthetic peptide TTB; A/Texas influenza antigen	vaccine	1-monopalmitoyl glycerol:CHOL:diacetyl phosphate (5:4:1 molar ratio), DCA (100 mg)	Thin-film hydration	/	[87]
A/Panama influenza antigen	vaccine	1-monopalmitoyl glycerol:CHOL:dicetyl phosphate (5:4:1 molar ratio), DCA 100 mg/mL	/	/	[88]
Influenza A antigen	vaccine	1-monopalmitoyl glycerol:CHOL:diacetyl phosphate (5:4:1 molar ratio), SDC (100 mg)	Thin-film hydration	/	[89]
Influenza antigen	vaccine	1-monopalmitoyl glycerol:CHOL:diacetyl phosphate (5:4:1 molar ratio), DCA (10 mM, 100 mM)	/	/	[90]
Influenza antigen	vaccine	Monopalmitoyl glycerol, CHOL, diacetyl phosphate, SDC (70–120 mM)	Design of Experiments	Monopalmitoyl glycerol:CHOL:diacetyl phosphate 5:4:1, SDC 100 mM	[91]
Hepatitis B surface antigen (HBsAg)	vaccine	Sorbitan tristearate:CHOL:diacetyl phosphate (7:3:1 molar ratio), SDC (100 mg)	Thin-film hydration	/	[92]
Hepatitis B surface antigen (HBsAg)	vaccine	Sorbitan tristearate:CHOL:modified dipalmitoyl phosphatidyl ethanolamine (7:3:1 molar ratio), SDC (100 mg), modified cholera toxin B	Thin film hydration	/	[93]
Tetanus Toxoid	vaccine	1-monopalmitoylglycerol, CHOL, diacetyl phosphate (5:4:1 molar ratio), SDC (100 mg)	Thin-film hydration	/	[94]
Tetanus Toxoid	vaccine	Span 80:CHOL:SDC (2:1:0.1 molar ratio), GM-OCM-DSPE (10%)	Thin-film hydration	/	[95]
Tetanus Toxoid	vaccine	Monopalmitoyl glycerol:CHOL:diacetyl phosphate (5:4:1), SDC (100 mM), xanthan gum (0.1% *w*/*v*)	Heating method (microwave method)	/	[96]

Abbreviations: CHOL, cholesterol; GM-OCM-DSPE, glucomannan-O-carboxymethyl-distearyl phosphatidyl ethanolamine; DCA, deoxycholic acid; SDC, sodium deoxycholate.

**Table 3:** Summary of studies investigating the peroral use of bilosomes.

Active Substance	Investigated Activity ^a^	Bilosome Composition	Formation Technique/Optimization	Optimal Formulation	Ref.
Epigallocatechin-gallate (EGCG)	/	Tween 40, CHOL, SC, SDC, STC, SDTC, SGC (20–160 mg)	Ethanol injection	CHOL:Tween 40 1:4 (*m*/*m*), SC:Tween 40 1:8 (*m*/*m*), EGCG:Tween 40 1:4 (*m*/*m*)	[102]
Lycopene	antibiotic	Span 60, CHOL (1:0.25 *w*/*w*), SC (0.01–0.04 molar concentration)	/	Span 60:CHOL 1:0.25 *w*/*w*, SC 0.02 molar concentration	[103]
Levofloxacin and Doxycycline hyclate	antibiotic	1-monopalmitoyl glycerol, CHOL, diacetyl phosphate (5:4:1), SDC	Melt method	/	[104]
Sertraline hydrochloride (SER)	antidepressive	Span 60, CHOL (Span 60:CHOL 1:1, 7:1 molar ratio), SDC (SER:SDC 0.25, 0.5 molar ratio)	Thin-film hydration/2^3^ full factorial design	Span:CHOL 1 molar ratio, SER:SDC 0.5 molar ratio	[105]
Apigenin	antidiabetic	CHOL (10–30%), Span 60 (50–70%), SDC (10–20%)	Thin-film hydration/3^3^ Box-Behnken design	CHOL 15.5%, Span 60 70.2%, SDC 12.4%	[106]
Berberine	antidiabetic	Soy PC (0.03–0.06 molar concentration), CHOL (10–20 mg), SDC (15–30 mg)	Thin-film hydration/Central composite design	Soy PC 0.06 molar concentration, CHOL 15.2 mg, SDC 25 mg	[107]
Insulin	antidiabetic	Soya beans seed extract:palmitic acid:CHOL (0.25:1:1 *w*/*w*), SDC 0.5%; palmitic acid:CHOL (1:1 *w*/*w*), SDC 0.5%	Thin-film hydration	/	[108]
Insulin	antidiabetic	Soy PC, CHOL, deoxycholic acid-glycine, deoxycholic acid-glytamil methylester, N α-deoxycholyl-L-lysyl-methylester	Reverse phase evaporation	/	[109]
Quercetin	antidiabetic	Soy PC:SDC (4:1), lactoferrin (5–40 mg/mL)	Thin-film hydration/	Soy PC:SDC 4:1, lactoferrin 30 mg/mL	[110]
Eprosartan mesylate	antidiabetic (diabetic neuropathy)	Soy PC, SDC (3:1, 4:1, 5:1, 6:1, 7:1. 9:1 ratio)	Thin-film hydration	Soy PC:SDC 4:1	[111]
Carvedilol	antihypertensive	Span 60:CHOL 4:1 molar ratio, SC, STC (20, 30%)	Thin-film hydration	SC 20%, STC 30%	[112]
Diclofenac sodium	anti-inflammatory	PC (0.5–1.5%), CHOL (0.1–0.5%), SDC (0.5–1.5%), Pluronic F127 (0.3–0.7%)	Thin-film hydration/3^4^ Box-Behnken design	PC 1% *w*/*v*, CHOL 0.3% *w*/*v*, SDC 1% *w*/*v*, Pluronic F127 0.5% *w*/*v*	[113]
Apigenin	antibiotic, cytotoxic	CHOL, PC, SDC (5, 10% *w*/*v*), Tween 80 (5, 10% *w*/*v*), SDC:Tween 80 (5, 10 % *w*/*v*), Chitosan (0.25, 0.5 % *w*/*v*)	Solvent-evaporation	SDC:Tween 80 10 % *w*/*v*, Chitosan 0.25%	[114]
Risedronate	antiosteoporotic	Soy PC:BS (SDC, STC, SGC):CHOL 4:1:0, 4:1:1, staerylamine, 1,2-dioleoyl-3-trimethyl ammonium propane	Reversed-phase evaporation/Thin-film hydration	/	[28]
Resveratrol	antioxidant, anti-inflammatory	Soy PC:CHOL:SDC (4:1:0, 4:1:1 molar ratio)	Thin-film hydration	Soy PC:CHOL:SDC 4:1:1 molar ratio, 3 extrusion cycles, drug concentration 10 mg/mL, pH 3	[77]
Torularhodin	antioxidant, anti-inflammatory	Lecithin, Tween 80, SDC	Thin-film hydration	/	[115]
Silymarin	antioxidant, hepatoprotective	Soy PC, SDC, SC, STC (Soy PC:BA 4:1)	Thin-film hydration	Soy PC:STC 4:1	[116]
Luteolin	antioxidant, antibiotic, cytotoxic	CHOL (1–5%), Span 60 (100–300 mg), SDC (0.15–0.35 mg), PEG 2000 (1–4 mg)	Thin-film hydration/3^4^ Box-Behnken design	CHOL (25 mg), Span 60 45 mg, SDC 12.5 mg, PEG 2000 30 mg	[117]
Quercetin	antioxidant, antibiotic, cytotoxic	Lipid (4–6%), Span 60 (3–7%), SDC (4–12%), Chitosan (0.2–0.3%)	Solvent evaporation method/3^3^ Box-Behnken design	Lipid 4%, Span 60 7%, SDC 8%, Chitosan 0.2%	[118]
Acyclovir	antiviral	CHOL (7.5–12.5 mg), Span 60, Tween 60 (1:2 ratio, 40–60 mg), SGC (5–10 mg)	Thin-film hydration/3^3^ Box-Behnken design	CHOL 10 mg, surfactant 50 mg, SGC 7.5 mg	[119]
Resveratrol	antiviral	Span 60, CHOL, SGC, SDC, Brij 20, Brij 72 (15 mg, 30 mg)	Ethanol injection/2^3^ full factorial design	SDC, Brij 20 15 mg	[120]
Sofosbuvir	antiviral	PC, Span 60 (Span 60:drug 1–5 ratio) STC (STC:Span 60 1–10 ratio), galactose	Thin- film hydration/ Central composite design	Span 60:drug 1:1 *w*/*w*, STC:Span 60 10:1 *w*/*w*	[121]
Piperine	antiviral, anti-inflammatory	Span 65, Brij 72, Brij 78, CHOL (Surfactant:CHOL 9:1, 1:1) SDC (1–5%)	Thin-film hydration/3^2^∙2^1^ Full factorial design	Brij72:CHOL 9:1, SDC 1%	[122]
Curcumin	cytotoxic	STC, SC (1%, 5% *w*/*w*), Span 60:CHOL (1:1, 5:1, 9:1 ratio), D-alpha-tocopheryl polyethylene glycol succinate (10, 20, 40 and 80 mg)	Thin-film hydration/3^1^∙2^2^ factorial design	STC 5% *w*/*w*, Span 60:CHOL 5:1, D-alpha-tocopheryl polyethylene glycol succinate 40 mg	[123]
Icariin, Melittin (MEL)	cytotoxic	CHOL, Span 20 (CHOL:Span 20 2, 4 molar ratio), SDC (0.25, 0.5 mM), MEL (1, 5% *w*/*w*)	Thin-film hydration/Box-Behnken design	CHOL:Span 20 2 molar ratio, SDC 0.25 mM, MEL 1.14% *w*/*w*	[124]
Piceatannol	cytotoxic	CHOL, Span 20 (1:2, 1:3, 1:4 molar ratio), SDC (0.25–0.5 mM), zein (5–10% *w*/*w*)	Thin-film hydration/3^3^ Box-Behnken design	CHOL:Span 1:3.977, SDC 0.435 mM, zein 7.052% *w*/*w*	[125]
Pitavastatin	cytotoxic	Soy PC, SDC, SC, STC (SPC:BS 2:1, 4:1, 6:1), lactoferrin (20–200 mg/mL)	Thin-film hydration/2^3^ Asymmetrical factorial design	Soy PC:SDC 4:1, lactoferrin 30 mg/ml	[126]
Psoralidin	cytotoxic	PC:CHOL:Span 60 1:0.4:0.2 molar ratio, SDC (0.125–0.5 mM), Chitosan 0.125–0.25% *w*/*v*	Thin-film hydration	SDC 0.25 mM, Chitosan 0.25 % *w*/*v*	[127]
Rolipram	cytotoxic	Brij 97, STC (1:1, 1:4)	Thin-film hydration	/	[128]
Silymarin	cytotoxic	Tween 20, Span 60 (100–300 mg), CHOL (10–20 mg), SDC (50–150 mg), Dextrose 60 1%, Dextrose 40 1%	Thin-film hydration/3^3^ Box Behnken design	/	[129]
Sulfated polysaccharide-protein complexes of *Enteromorpha intestinalis*	cytotoxic	CHOL, Span 40, Span 65 (CHOL:Span 1:5 molar ratio), SC, SDC, STDC	Thin-film hydration	Span 65, SC	[130]
Chrysin	hepatoprotective	CHOL (10–40 mg) Lecithin (100–200 mg), SDC (10–40 mg)	Thin-film hydration/Fractional factorial design	CHOL 20 mg, Lecithin 100 mg, SDC 20 mg	[131]
Progesterone	hormone	PC (0.75–1.25% *w*/*v*), CHOL (0.15–0.45% *w*/*v*), SDC (0.2–0.3% *w*/*v*)	Thin-film hydration/3^3^ Box-Behnken design	PC 1.25% *w*/*v*, CHOL 0.15% *w*/*v*, SDC 0.29% *w*/*v*	[132]
Cyclosporin A (CyA)	immunosuppressive	CHOL, Soy PC (2–6%), SDC (Soy PC:SDC 3:1–9:1) gelatin (0–20%)	Thin-layer hydration with high-pressure homogenization	Soy PC 6%, Soy PC:SDC 3:1, CyA 2 mg/mL, gelatin 10%	[133]
Berberine and Curcumin (CUR)	NAFLD therapy	SPC, CHOL, octadecylamine, SDC, CUR (20:2:1:3:1 mass ratio), diethylaminoethyl dextran (0.13–0.52 mg/mL)	Thin-film hydration	diethylaminoethyl dextran 0.39 mg/mL	[134]
*Bacopa monnieri*	nootropic	Soy PC (400–1600 mg), SDC (5–10 mg)	Thin-film hydration	Soy PC 1600 mg, SDC 10 mg	[135]

^a^ In alphabetical order. Abbreviations: BA, bile acid; BS, bile salt; CHOL, cholesterol; PC, phosphatidylcholine; SC, sodium cholate; SDC, sodium deoxycholate; SGC, sodium glycocholate; STC, sodium taurocholate; SDTC, sodium deoxytaurocholate.

**Table 4:** Summary of studies investigating the intranasal use of bilosomes.

Active Substance	Investigated Activity ^a^	Bilosome Composition	Formation Technique/Optimization	Optimal Formulation	Ref.
Luteolin	anti-Alzheimer’s disease	Lipoid S100:CHOL (2:1, 4:1 molar ratio), Span 60 (100, 200 mg), SDC (10, 25 mg)	Thin-film hydration/2^3^ factorial design	CHOL:Lipoid S100 2:1 molar ratio, Span 60 100 mg, SDC 25 mg	[140]
Resveratrol with paramagnetic iron oxide	anti-Alzheimer’s disease	Span 60:CHOL (2:1, 1:1, 1:2 molar ratio), SDC (10 mg), chitosan (0.05–0.2% *w*/*v*)	Thin-film hydration	Span 60:CHOL 1:1 molar ratio, SDC 10 mg, chitosan 0.1% *w*/*v*	[141]
Doxylamine Succinate, Pyridoxine hydrochloride	antiemetic (gestational nausea and vomiting)	Soy PC (1–5% *w*/*v*), CHOL (15–50 mg), SC (5–10% *w*/*w*), poloxamer 407:poloxamer 188:carbopol 971P (20:10:0.2% *w*/*w*)	Thin-film hydration/3^3^ Box-Behnken design	Soy PC 2.09% *w*/*v*, CHOL 31.71 mg, SC 6.37% *w*/*w*	[142]
Budesonide	anti-inflammatory	CHOL, Phospholipoin 80H, L-α phosphatidylcholine, Lipoid S45 (0.0965 g), Span 60 (1.1965 g), SC, SDC (0.215 g)	Thin-film hydration/Mixed-factorial design	CHOL 0.0965 g, SC 0.215 g, budesonide 10 mg	[143]
Zolmitriptan (Migraine)	antimigraine	CHOL:Span 40 (1:1–1:9 molar ratio, 100–300 mg), SDC (5–15 mg), HPMC 0.5% *w*/*v*, poloxamer 407 17% *w*/*v*	Thin-film hydration/3^3^ Box-Behnken design	CHOL:Span 40 1:7.7 molar ratio 255 mg, SDC 5 mg	[144]

^a^ In alphabetical order. Abbreviations: PC, phosphatidylcholine; CHOL, cholesterol; SC, sodium cholate; SDC, sodium deoxycholate; HPMC, hydroxypropyl methylcellulose.

**Table 5:** Summary of studies investigating the ocular use of bilosomes.

Active Substance	Investigated Activity ^a^	Bilosome Composition	Formation Technique/Optimization	Optimal Formulation	Ref.
Ciprofloxacin	antibiotic	CHOL (10 mg, 30 mg), Span 60 (40 mg, 60 mg), SDC (15 mg, 25 mg), HPMC K100 M (0.2–1% *w*/*v*), Carbopol-934P (1–1.8% *w*/*v*)	Thin-film hydration/Box-Behnken design	CHOL 35 mg, Span 60 65 mg, SDC 20 mg, HPMC K100M 0.6% *w*/*v*, Carbopol 934P 1.4% *w*/*v*	[149]
Moxifloxacin	antibiotic	Span 60 (30–60 mg), CHOL (15%), Cremophor EL (7–15 mg), SDC (15–30 mg), chitosan (0.35%), sodium alginate (0.1–0.5%)	Thin-film hydration/3^3^ Box-Behnken design	CHOL 15%, Span 60 30 mg, Cremophor EL 13 mg, SDC 18 mg, CH 0.35%, SA 0.4%	[150]
Natamycin	antifungal	Span 60:CHOL (2:1, 1:1, 1:2 molar ratio), STC (10 mg, 21 mg), gellan gum (20–40 mg), xanthan gum (20–40 mg)	Thin-film hydration	Span 60:CHOL 1:1 molar ratio, STC 10 mg, gellan gum 30 mg	[151]
Terconazole	antifungal	Span 60, CHOL, STC (10 mg, 20 mg), Cremophor EL, Cremophor RH 40 (5 mg, 10 mg)	Ethanol injection/2^3^ factorial design	Span 60, CHOL, STC 10 mg, Cremophor EL 5 mg	[152]
Terconazole	antifungal	Span 60 (60–100 mg), CHOL (0–20 mg), SDC (0–10 mg), Labrafil^®^ M 2125 CS:Tween^®^ 80:Transcutol^®^ HP (20:50:30)	Thin-film hydration/Box-Behnken design	Span 60 73.59 mg, CHOL 1.28 mg, SDC 3.11 mg, Labrafil^®^ M 2125 CS:Tween^®^ 80:Transcutol^®^ HP (20:50:30)	[153]
Acetazolamide	antiglaucoma	Span 60, CHOL, SC, SDC, STC, STGC (Span 60:CHOL:BA 1:1:0.1, 1:1:0.2 molar ratio)	Thin-film hydration	Span 60:CHOL:SDC 1:1:0.2 molar ratio	[154]
Agomelatine	antiglaucoma	PC, SC, SDC, STC (PC:BA 2:1, 4:1), hyaluronic acid (0%, 0.5%)	Ethanol injection/-optimal design	PC:SC 2:1 ratio, hyaluronic acid 0.26%	[155]
Betaxolol hydrochloride	antiglaucoma	Soy PC, Span 60, CHOL (4:1:1 ratio), Cremophor EL (5 mg, 10 mg) SDC, STC (0.25%, 0.75% *w*/*v*)	Ethanol injection/2^3^ factorial design	Soy PC, Span 60, CHOL (4:1:1 ratio), Cremophor EL 10 mg, SDC 0.75% *w*/*v*	[156]
Brinzolamide	antiglaucoma	Span 60, CHOL, SDC, STC, Kolliphor RH40, Tween 80	Ethanol injection	Span 60:CHOL:STC:Tween 80 5:5:2:1 ratio	[157]

^a^ in alphabetical order. Abbreviations: BA, bile acid; CHOL, cholesterol; SC, sodium cholate; SDC, sodium deoxycholate; STC, sodium taurocholate, HPMC, hydroxypropyl methylcellulose.

**Table 6:** Summary of studies investigating the buccal use of bilosomes.

Active Substance	Investigated Activity ^a^	Bilosome Composition	Formation Technique/Optimization	Optimal Formulation	Ref.
Insulin	antidiabetic	Soy lecithin, SC, STC, SGC, SDGC, SDTC (85:15 *w*/*w*%)	Thin-film hydration	Soy lecithin, SDGC 85:15 *w*/*w*%	[162]
Carvedilol	Antihypertensive	Soy PC (1–3% *w*/*w*), CHOL (0–30% *w*/*w*), diacetyl phosphate (5% *w*/*w* of total lipid), SDC (25 mg), CMC:HPC (1:1)	Thin-film hydration	Soy PC 3% *w*/*w*, CHOL 30% *w*/*w*, diacetyl phosphate 5% of total lipid, SDC 25 mg, CMC:HPC 1:1	[163]
Vardenafil	PDE-5 inhibitor	Soy PC (1–3 molar concentration), CHOL (0–30 molar concentration), SDC (10–30 mg), HPMC:CMC (50:50 ratio)	Thin-film hydration/3^3^ Box-Behnken design	Soy PC 2.98%, CHOL 29.4%, SDC 17.25 mg	[164]

^a^ In alphabetical order. Abbreviations: PC, phosphatidylcholine; CMC, carboxymethyl cellulose CHOL, cholesterol; SC, sodium cholate; SDC, sodium deoxycholate; SGC, sodium glycocholate; STC, sodium taurocholate; SDGC, sodium deoxyglycocholate; SDTC, sodium deoxytaurocholate; HPC, hydroxypropyl cellulose; HPMC, hydroxypropyl methylcellulose.

**Table 7:** Summary of a study investigating vaginal use of bilosomes.

Active Substance	Investigated Activity	Bilosome Composition	Formation Technique/Optimization	Optimal Formulation	Ref.
Fenticonazole nitrate	antifungal	limonene:citral (1:1, 1:2, 1:3 *w*/*w*), SDC (10–30 mg), L-α-PC 100 mg, ethanol (5–15% *v*/*v*), HPMC	Thin-film hydration/3^3^ Box-Behnken design	limonene:citric 1:3 *w*/*w*, SDC 10 mg, ethanol 11.26% *v*/*v*	[168]

Abbreviations: PC, phosphatidylcholine; SDC, sodium deoxycholate; HPMC, hydroxypropyl methylcellulose.

## Data Availability

No new data were created or analyzed in this study. Data sharing is not applicable to this article.
